# Combined Influence of Depressive Symptoms and Estimated Glomerular Filtration Rate on Cognition Decline in US Adults

**DOI:** 10.1002/brb3.70997

**Published:** 2025-11-26

**Authors:** Yuxin Yang, Haoxiang Hu, Tian Lv, Jie Li, Yue Yang, Shiqin Chen, Yuping He, Qinwen Fei

**Affiliations:** ^1^ College of Medical Shaoxing University Shaoxing Zhejiang China; ^2^ Postgraduate Training Base Alliance of Wenzhou Medical University, Wenzhou People's Hospital Wenzhou China; ^3^ Department of Neurology Zhuji Affiliated Hospital of Wenzhou Medical University Zhuji China; ^4^ Wenzhou Medical University Lishui Hospital, Lishui People's Hospital Lishui Zhejiang China; ^5^ Department of Neurology Yuhuan Second People's Hospital Yuhuan Zhejiang China; ^6^ Department of Geriatrics Zhuji Affiliated Hospital of Wenzhou Medical University Zhuji China

**Keywords:** cognition decline, depressive symptoms, estimated glomerular filtration rate, NHANES

## Abstract

**Background:**

Cognitive decline is common in older adults and may be influenced by both depressive symptoms and kidney function.

**Methods:**

We analyzed 2650 participants aged ≥ 60 years from NHANES 2011–2014. Depressive symptoms were defined as PHQ‐9 ≥ 10, and eGFR was calculated using the CKD‐EPI equation. Cognitive decline was defined as the lowest quartile of a composite score from CERAD, AFT, and DSST tests. Weighted logistic regression, restricted cubic spline, and mediation analyses were applied.

**Results:**

Depressive symptoms (OR = 3.14, 95% CI: 1.91–5.19) and reduced eGFR < 60 mL/min/1.73 m^2^ (OR = 2.13, 95% CI: 1.25–3.61) were independently associated with a higher risk of cognitive decline. Participants with both depressive symptoms and low eGFR had the greatest risk (OR = 11.74, 95% CI: 4.02–34.28). Nonlinear analysis showed an L‐shaped association between eGFR and cognition, and mediation analysis suggested depressive symptoms accounted for 15% of the effect of eGFR on cognition. Sex‐stratified results indicated stronger combined effects in females.

**Conclusions:**

Both depressive symptoms and reduced eGFR are independent risk factors for cognitive decline, and their coexistence markedly amplifies risk, particularly in females. These findings highlight the importance of integrated screening and intervention for mental and renal health in older adults.

## Graphical abstract

Depressive symptoms and reduced renal function were independently associated with dementia in US adults, and their coexistence markedly amplified risk, underscoring the importance of integrating mental and renal health in dementia prevention.

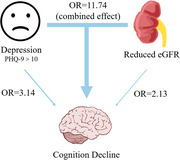

## 1 Introduction

With the rise in global aging, cognitive decline has emerged as a prevalent neurodegenerative disorder affecting a growing number of older individuals. This condition significantly compromises quality of life while imposing heavy economic costs on families and society (Prince et al. 2013). The development of cognitive decline involves a complex interplay of factors, including genetics, lifestyle, and cardiovascular health (Livingston et al. 2017). Previous research has shown that depression notably increases the risk of developing dementia later in life (Steffens 2021). As a common mental health issue in older adults, depression is both an independent risk factor and an early warning sign of dementia (Piras et al. 2021). Potential mechanisms include disrupted cerebral blood flow, heightened inflammatory activity, and neurotransmitter imbalances (Huang et al. 2024). Additionally, individuals with depression often have poor lifestyle habits, including low physical activity, inadequate nutrition, and sleep disorders, all of which contribute to a higher risk of cognitive decline. Depression may also intensify chronic conditions like diabetes and cardiovascular diseases, further raising dementia risk (van Sloten et al. 2020; Mukherjee et al. 2024).

In parallel, renal dysfunction, especially reduced estimated glomerular filtration rate (eGFR), has been linked to both cognitive impairment and dementia (Viggiano et al. 2020; Singh‐Manoux et al. 2022). Cognitive impairment in renal dysfunction is attributed to mechanisms such as toxin buildup, increased inflammation, electrolyte disturbances, and vascular damage (Wagner et al. 2024). Drew et al. (2019) found that cognitive performance declines with worsening renal function, as reflected in reduced glomerular filtration rate (eGFR). Additionally, chronic kidney disease (CKD) is often accompanied by cardiovascular and metabolic complications, which may jointly elevate dementia risk through various pathological pathways (Jankowski et al. 2021). The polypharmacy commonly required to manage CKD can further negatively affect the central nervous system, aggravating cognitive decline (Kimura et al. 2023).

Despite well‐documented independent associations between depression and renal dysfunction with cognitive decline, their potential combined impact and interrelated mechanisms remain insufficiently explored. Both conditions can contribute to vascular and metabolic abnormalities, chronic inflammation, and lifestyle deterioration, all of which may accelerate cognitive deficits (Huang et al. 2024; Wagner et al. 2024). This study, therefore, leverages the NHANES database to investigate the independent and combined effects of depressive symptoms and reduced eGFR on cognitive decline. In addition, we further explore sex‐specific differences and potential mediating pathways. By simultaneously considering psychosocial and renal factors, this study provides a more integrative perspective and offers novel insights for targeted prevention and management strategies for cognitive impairment.

## 2 Methods

### 2.1 Study Population

**FIGURE 1:**
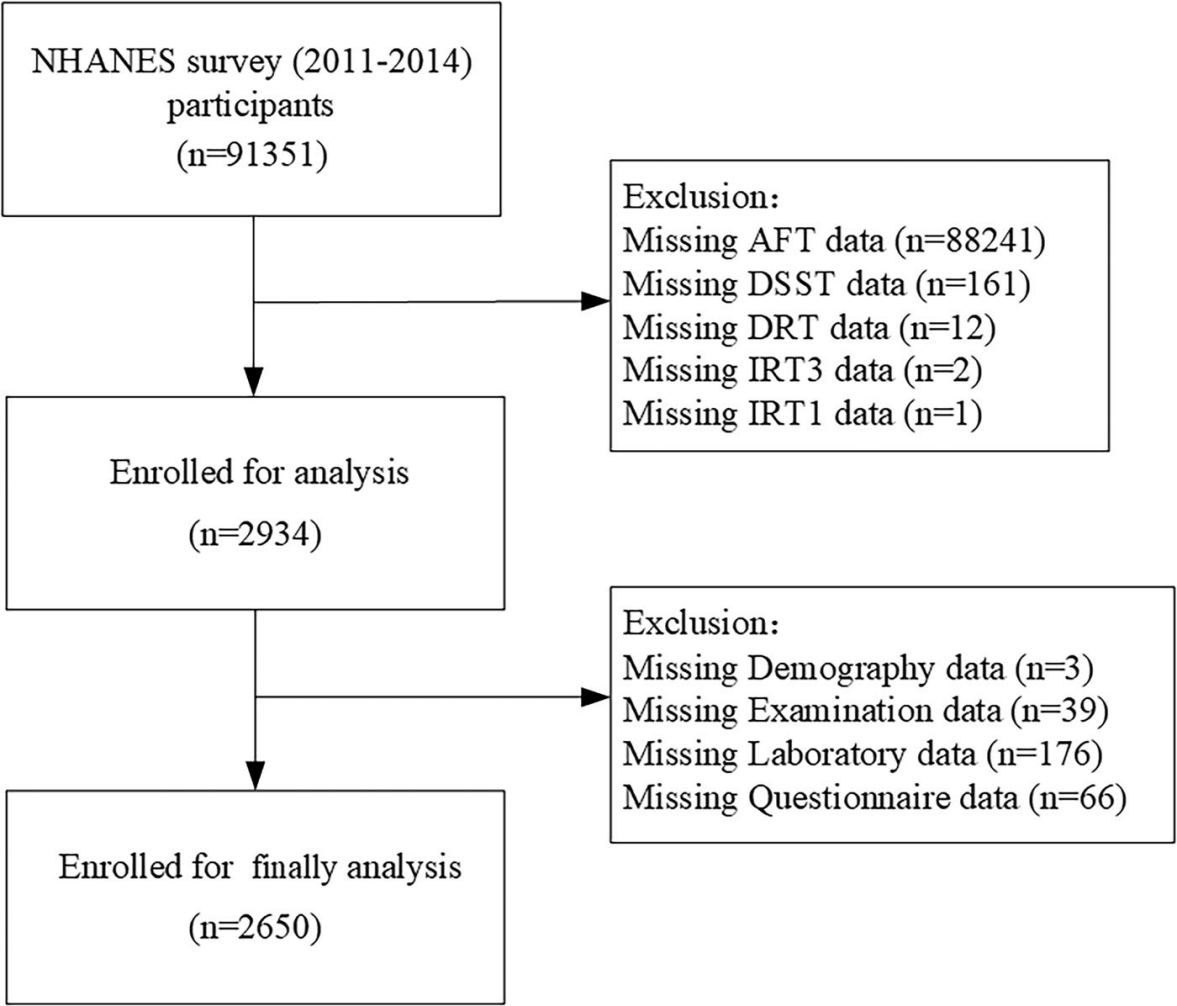
Flow chart of the study population.

This research utilized data from NHANES, a health and nutrition survey conducted by the CDC that is cross‐sectional and nationally representative. Participants were drawn from the 2011 to 2012 and 2013 to 2014 cycles. We restricted the analysis to adults aged ≥ 60 years who had completed all three cognitive tests ([Consortium to Establish a Registry for Alzheimer's Disease] CERAD Word Learning Test, Animal Fluency Test [AFT], and Digit Symbol Substitution Test [DSST]), with available Patient Health Questionnaire‐9 (PHQ‐9) scores and eGFR data. Exclusions were made for participants with missing data on key variables. Specifically, missing values were observed for AFT scores (*n* = 88,241), DSST scores (*n* = 161), Delayed Recall Test (DRT) scores (*n* = 12), Immediate Recall Test (IRT) scores (*n* = 3), education level (*n* = 3), neutrophil count (*n* = 106), smoking (*n* = 2), alcohol use (*n* = 52), sleep duration (*n* = 6), bilirubin (*n* = 70), BMI (*n* = 39), and PHQ‐9 (*n* = 6). After these exclusions, 2650 participants remained for the final analysis (Figure 1). Because NHANES is a nationally representative survey with a fixed sample size, no a priori sample size calculation was performed. Instead, all eligible participants with complete data were included, which ensured sufficient statistical power for the present analyses.

### 2.2 Definition of Independent Variables

Depressive symptoms were assessed using the PHQ‐9, a standardized self‐report instrument included in the NHANES mental health module (X. Liu et al. 2023; Levis et al. 2019). Scores range from 0 to 27, with higher values indicating greater symptom severity. As a screening rather than diagnostic tool, the PHQ‐9 identifies individuals with clinically relevant depressive symptoms rather than providing a formal diagnosis. A cutoff score of ≥ 10 was applied, in line with established NHANES protocols and supported by large‐scale validation studies (Patel et al. 2019; Manea et al. 2012). The eGFR was calculated using the CKD Epidemiology Collaboration (CKD‐EPI) equation, which was categorized into three levels: < 60, 60–90, and > 90 mL/min/1.73 m^2^ (Levis et al. 2019).

### 2.3 Outcome Variable

This study utilized cognitive tests from the National Health and Nutrition Examination Survey, conducted from 2011 to 2014 on individuals aged 60 and above. These assessments are part of the standardized NHANES cognitive evaluation protocol and have been widely used in population‐based studies to examine multiple cognitive domains (Dong et al. 2020; Fan et al. 2021). These tests included the CERAD Word Learning Subtest, the AFT, and DSST (Dong et al. 2020). The CERAD Subtest assessed memory through three trials of learning 10 words followed by a delayed recall, with scores ranging from 0 to 10 for each trial and a total score summing all trials (Fan et al. 2021). The AFT tested verbal fluency, where participants named as many animals as possible in 1  min, with correct responses contributing to the total score. The DSST measured processing speed, attention, and working memory by having participants match symbols to numbers in 133 boxes within 2 min. A composite cognitive score was calculated by summing the results of these tests, and participants in the lowest quartile of scores were identified as cognitively impaired (Guo et al. 2022).

### 2.4 Covariates

Covariates included in this analysis were age, sex, race, and educational level; smoking status, alcohol consumption, sleep duration, BMI, and histories of hypertension and hyperlipidemia; uric acid and bilirubin, which have been reported as potential protective factors against dementia (Z. Zhou et al. 2021; Gao et al. 2024), total protein, which has been associated with cognitive outcomes in older adults (Song et al. 2023), serum creatinine (for eGFR calculation), and neutrophil‐to‐lymphocyte ratio (NLR) as an index of systemic inflammation (Hung et al. 2023). Sex was classified as male or female, while race categories were non‐Hispanic White, non‐Hispanic Black, and other races. Educational attainment was grouped into below college and college or higher. Smoking status was categorized as never, former, and current smoker, and alcohol intake as never, former, mild, moderate, and heavy. Sleep duration was recorded in hours per night. BMI was calculated as weight (kg) divided by height squared (m^2^). Hypertension was defined as systolic blood pressure (SBP) ≥ 140 mmHg, diastolic blood pressure (DBP) ≥ 90 mmHg, or self‐reported hypertension (Carey and Whelton 2018). Hyperlipidemia was diagnosed using criteria such as total cholesterol ≥ 200 mg/dL, low‐density lipoprotein (LDL) ≥ 130 mg/dL, high‐density lipoprotein (HDL) < 40 mg/dL for males or < 50 mg/dL for females, triglycerides ≥ 150 mg/dL, or lipid‐lowering medication use (Kuwabara et al. 2018). Protein and bilirubin levels were obtained from lab results. NLR was calculated as the ratio of neutrophil count to lymphocyte count (Chen et al. 2023), and eGFR was estimated using the CKD‐EPI formula (Kidney Disease: Improving Global Outcomes (KDIGO) Glomerular Diseases Work Group 2021). These covariates were included to minimize confounding effects.

### 2.5 Statistical Analysis

This study followed NHANES analytic protocols and accounted for the complex survey design by applying mobile examination center (MEC) weights. Continuous variables were expressed as weighted mean ± SE and categorical variables as weighted percentages. Between‐group differences were evaluated using survey‐weighted *t*‐tests or nonparametric tests for continuous variables and Chi‐square tests for categorical variables.

Multivariable logistic regression was performed to examine the independent associations of depressive symptoms and eGFR with cognitive decline. Odds ratios (ORs) and 95% confidence intervals (CIs) were reported. Three models were constructed: a crude model without adjustment, Model 1 adjusted for age, sex, race, and education, and Model 2 further adjusted for uric acid, NLR, BMI, protein, bilirubin, sleep duration, smoking status, alcohol use, hypertension, and hyperlipidemia. To assess the robustness of the findings, we additionally conducted a sensitivity analysis using multivariable linear regression, in which the composite cognitive score was treated as a continuous outcome and the same set of covariates as in Model 2 were included. Results were consistent with those of logistic regression (Table S2). Model fit was evaluated using Akaike Information Criterion (AIC), and detailed values are provided in Table S3. Restricted cubic spline (RCS) models were applied to evaluate potential nonlinear relationships between PHQ‐9 score, eGFR, and the risk of cognitive decline. Subgroup and stratified analyses were also performed according to age and sex.

Mediation analysis was used to test whether depressive symptoms mediated the association between eGFR and cognitive impairment, with direct and indirect effects estimated using a bootstrap approach with 5000 resamples. We evaluated potential interactions in logistic regression models. Specifically, we tested multiplicative and additive interactions between depressive symptoms (PHQ‐9) and renal function (eGFR). In addition, we examined higher‐order two‐way interactions between key covariates (age × PHQ‐9, age × eGFR, sex × PHQ‐9, sex × eGFR). Additive interaction was assessed using Relative Excess Risk due to Interaction (RERI), Attributable Proportion due to Interaction (AP), and Synergy Index (SI) (Knol et al. 2011). All analyses were performed using R (version 4.3.2). To account for multiple comparisons, *p* values were adjusted using the Benjamini–Hochberg false discovery rate (FDR) procedure, based on the total number of hypothesis tests within each analytic framework (regression, subgroup, and interaction analyses). Adjusted *p* values are reported in the tables. A two‐sided *p* < 0.05 was considered statistically significant.

## 3 Results

### 3.1 Baseline Characteristics

Of the 2650 participants in this study, 53.79% were female and 46.21% male. A total of 653 participants were classified as cognition decline. The average age of the sample was 69.12 years, participants without cognitive deterioration averaged 68.42 years, and cognition decline participants averaged 73.28 years. The cognition decline group had significantly higher neutrophil, protein, and creatinine levels, while showing lower NLR, eGFR, and cognitive scores (IRT, DRT, DSST, AFT) (all *p* < 0.05). There were no significant differences in sex, lymphocyte counts, uric acid, bilirubin, sleep duration, BMI, smoking status, or hyperlipidemia prevalence (Table 1).

**TABLE 1:** Descriptive baseline characteristics of participants.

Variable	Total (*n* = 2650)	Without cognition decline (*n* = 1997)	With cognition decline (*n* = 653)	*p* value
Age	69.12 (0.20)	68.42 (0.19)	73.28 (0.48)	< 0.0001
Sex				0.651
Female	1351 (53.79)	1061 (54.00)	290 (52.55)	
Male	1299 (46.21)	936 (46.00)	363 (47.45)	
Education				< 0.0001
< high	651 (15.56)	290 (10.45)	361 (46.03)	
College	1371 (62.47)	1226 (68.27)	145 (27.89)	
High	628 (21.97)	481 (21.28)	147 (26.08)	
Race				< 0.0001
Black	596 (7.82)	394 (6.20)	202 (18.07)	
Other	761 (11.89)	515 (9.88)	246 (23.85)	
White	1293 (80.30)	1088 (84.02)	205 (58.08)	
Neutrophil (%)	59.79 (0.27)	59.59 (0.29)	60.92 (0.54)	0.034
Lymphocyte (%)	28.03 (0.26)	28.15 (0.28)	27.33 (0.52)	0.151
NLR	2.51 (0.04)	2.47 (0.04)	2.71 (0.10)	0.027
Uric acid	5.61 (0.04)	5.60 (0.04)	5.701 (0.07)	0.22
Protein (g/L)	69.38 (0.14)	69.23 (0.15)	70.24 (0.27)	< 0.001
Bilirubin	0.69 (0.01)	0.69 (0.01)	0.69 (0.02)	0.77
Sleep hour (h)	7.16 (0.02)	7.15 (0.03)	7.21 (0.08)	0.483
PHQ‐9 score	2.84 (0.12)	2.57 (0.11)	4.46 (0.38)	< 0.0001
Cognition tests score				
IRT	19.79 (0.23)	20.68 (0.22)	14.50 (0.29)	< 0.0001
DRT	6.28 (0.10)	6.68 (0.09)	3.89 (0.12)	< 0.0001
DSST	52.55 (0.58)	56.87 (0.47)	26.82 (0.64)	< 0.0001
AFT	18.18 (0.18)	19.24 (0.19)	11.89 (0.16)	< 0.0001
Total	96.81 (0.90)	103.468 (0.76)	57.09 (0.63)	< 0.0001
Creatinine	0.99 (0.01)	0.95 (0.01)	1.24 (0.08)	0.001
PHQ‐9 score	2.84 (0.12)	2.57 (0.11)	4.46 (0.38)	< 0.0001
eGFR (CKD‐EPI)	73.35 (0.37)	74.64 (0.41)	65.61 (0.91)	< 0.0001
BMI	29.05 (0.22)	29.11 (0.26)	28.68 (0.41)	0.425
Hypertension				< 0.0001
No	793 (34.03)	646 (36.32)	147 (20.35)	
Yes	1857 (65.97)	1351 (63.68)	506 (79.65)	
Smoke status				0.081
Former	1001 (39.09)	762 (39.46)	239 (36.86)	
Never	1306 (49.75)	994 (49.90)	312 (48.83)	
Now	343 (11.17)	241 (10.64)	102 (14.31)	
Alcohol status				< 0.0001
Former	730 (22.97)	490 (20.71)	240 (36.48)	
Heavy	195 (5.95)	137 (5.87)	58 (6.44)	
Mild	1053 (46.51)	886 (49.34)	167 (29.62)	
Moderate	264 (11.79)	214 (12.76)	50 (6.01)	
Never	408 (12.78)	270 (11.32)	138 (21.45)	
Hyperlipidemia				0.778
No	438 (15.85)	327 (15.78)	111 (16.28)	
Yes	2212 (84.15)	1670 (84.22)	542 (83.72)	

Abbreviations: AFT: Animal Fluency test; BMI: body mass index; CKD‐EPI: Chronic Kidney Disease Epidemiology Collaboration; DRT: Delayed Recall Test; DSST: Digit Symbol Substitution Test; eGFR: estimated Glomerular Filtration Rate; IRT: Immediate Recall Test; NLR: neutrophil–lymphocyte ratio; PHQ‐9: Patient Health Questionnaire.

### 3.2 Association Between Depressive Symptoms or eGFR and Cognition Decline Symptoms

Analyzing PHQ‐9 scores as a continuous variable revealed a 10% increase in cognition decline risk per 1‐point score increase in the crude model (OR = 1.10, 95% CI: 1.06–1.13, *p* < 0.001). This increased to 11% in Model 1 (OR = 1.11, 95% CI: 1.07–1.15, *p* < 0.001) and remained unchanged in Model 2 (OR = 1.11, 95% CI: 1.07–1.15, *p* < 0.001). When categorized, depressive symptoms (PHQ‐9 ≥ 10) significantly heightened cognition decline risk compared to those without signs of depression (PHQ‐9 < 10). The OR in the crude model was 2.90 (95% CI: 1.98–4.24, *p* < 0.0001). Model 1 adjusted the OR to 3.14 (95% CI: 2.08–4.75, *p* < 0.0001), with Model 2 yielding a similar result (OR = 3.14, 95% CI: 1.91–5.19, *p* < 0.001) (Table 2A).

**TABLE 2:** Multivariate logistic regression analyses for the associations between depressive symptoms, eGFR, and their combined effects on cognition decline.

(A) Association between depressive symptoms (PHQ‐9 score) and cognition decline						
Exposure	Crude model		Model 1		Model 2	
	95% CI	*p*	95% CI	*p*	95% CI	*p*/adjusted *p*
PHQ‐9 score	1.10 (1.06, 1.13)	< 0.0001	1.11 (1.07, 1.15)	< 0.0001	1.11 (1.07, 1.15)	< 0.001/< 0.001
Depressive symptoms						
No	Ref		Ref		Ref	
Yes	2.90 (1.98, 4.24)	< 0.001	3.14 (2.08, 4.75)	< 0.001	3.14 (1.91, 5.19)	< 0.001/< 0.001
*p* for trend		< 0.001		< 0.001		< 0.001

(B) Association between eGFR and cognition decline						
Exposure	Crude model		Model 1		Model 2	
	95% CI	*p*	95% CI	*p*	95% CI	*p*/adjusted *p*
eGFR (mL/min/1.73 m^2^)						
eGFR (CKD‐EPI)	1.74 (1.44, 2.09)	< 0.0001	1.42 (1.09, 1.83)	0.01	1.54 (1.13, 2.08)	0.01/0.01
> 90	Ref		Ref		Ref	
60–90	1.12 (0.80, 1.56)	0.49	0.95 (1.65, 1.38)	0.78	1.08 (0.72, 1.63)	0.68/0.68
< 60	3.02 (2.08, 4.38)	< 0.001	1.68 (1.05, 2.68)	0.03	2.13 (1.25, 3.61)	0.01/0.02
*p* for trend		< 0.001		0.01		0.01

(C) Combined effect of depressive symptoms and eGFR on cognition decline						
Exposure	Crude model		Model 1		Model 2	
	95% CI	*p*	95% CI	*p*	95% CI	*p*/adjusted *p*
Combined						
No depressive symptoms and eGFR > 90	Ref		Ref		Ref	
Depressive symptoms and eGFR > 90	3.40 (1.72, 6.70)	< 0.001	2.45 (1.47, 4.08)	0.001	2.60 (1.43, 4.73)	0.01/0.025
No depressive symptoms and 60 < eGFR < 90	1.23 (0.84, 1.79)	0.28	0.96 (0.64, 1.43)	0.83	1.07 (0.69, 1.64)	0.73/0.73
Depressive symptoms and 60 < eGFR < 90	2.71 (1.52, 4.85)	0.002	2.09 (1.09, 3.99)	0.03	2.62 (1.19, 5.77)	0.02/0.033
No depressive symptoms and eGFR < 60	3.06 (2.00, 4.67)	< 0.001	1.46 (0.90, 2.38)	0.12	1.90 (1.06, 3.39)	0.03/0.037
Depressive symptoms and eGFR < 60	11.41 (5.37, 24.24)	< 0.001	8.48 (3.01,23.90)	< 0.001	11.74 (4.02, 34.28)	< 0.001/0.005
*p* for trend		< 0.001		0.001		< 0.001

*Note*: Crude Model: unadjusted. Model 1: adjusted for included crude model, age, sex, education and race. Model 2: adjusted for all model 1 in addition to uric acid, NLR, protein, bilirubin, sleep hour, creatinine, estimated glomerular filtration rate, BMI, smoking status, alcohol status, hyperlipidemia and hypertension. PHQ‐9: Patient Health Questionnaire. Combined effects: the estimates for combined categories represent the risk associated with belonging to that joint exposure group compared with the reference group, and do not by themselves indicate statistical interaction.

For eGFR as a continuous variable, the crude model showed a strong association with cognition decline risk (OR = 1.74, 95% CI: 1.44–2.09, *p* < 0.001). After adjustments, the OR in Model 1 decreased to 1.42 (95% CI: 1.09–1.83, *p* = 0.01), and in Model 2, it was 1.54 (95% CI: 1.13–2.08, *p* = 0.01). When categorized into < 60, 60–90, and > 90 mL/min/1.73 m^2^, participants with eGFR < 60 mL/min/1.73 m^2^ had a significantly higher cognition decline risk compared to the reference group (eGFR > 90 mL/min/1.73 m^2^). In the crude model, the OR was 3.02 (95% CI: 2.08–4.38, *p* < 0.0001). Model 1 reported an OR of 1.68 (95% CI: 1.05–2.68, *p* = 0.03), and Model 2 showed a significantly increased risk (OR = 2.13, 95% CI: 1.25–3.61, *p* = 0.01) (Table 2B). Model fit improved across successive adjustments, with AIC decreasing from 1695 (m0) to 1393 (m2) for eGFR–cognition models and from 1745 (m0) to 1368 (m2) for PHQ‐9–cognition models. (Table S3)

In sensitivity analyses treating the composite cognitive score as a continuous variable, both depressive symptoms and lower eGFR remained significantly associated with poorer cognition, consistent with the primary logistic regression results (Table S2). Similarly, in linear regression treating cognition as a continuous outcome (Table S2), AIC decreased from 8400 (m0) to 6989 (m2) for PHQ‐9 models and from 8351 (m0) to 7059 (m2) for eGFR models, further supporting the robustness of the associations (Table S3).

**FIGURE 2:**
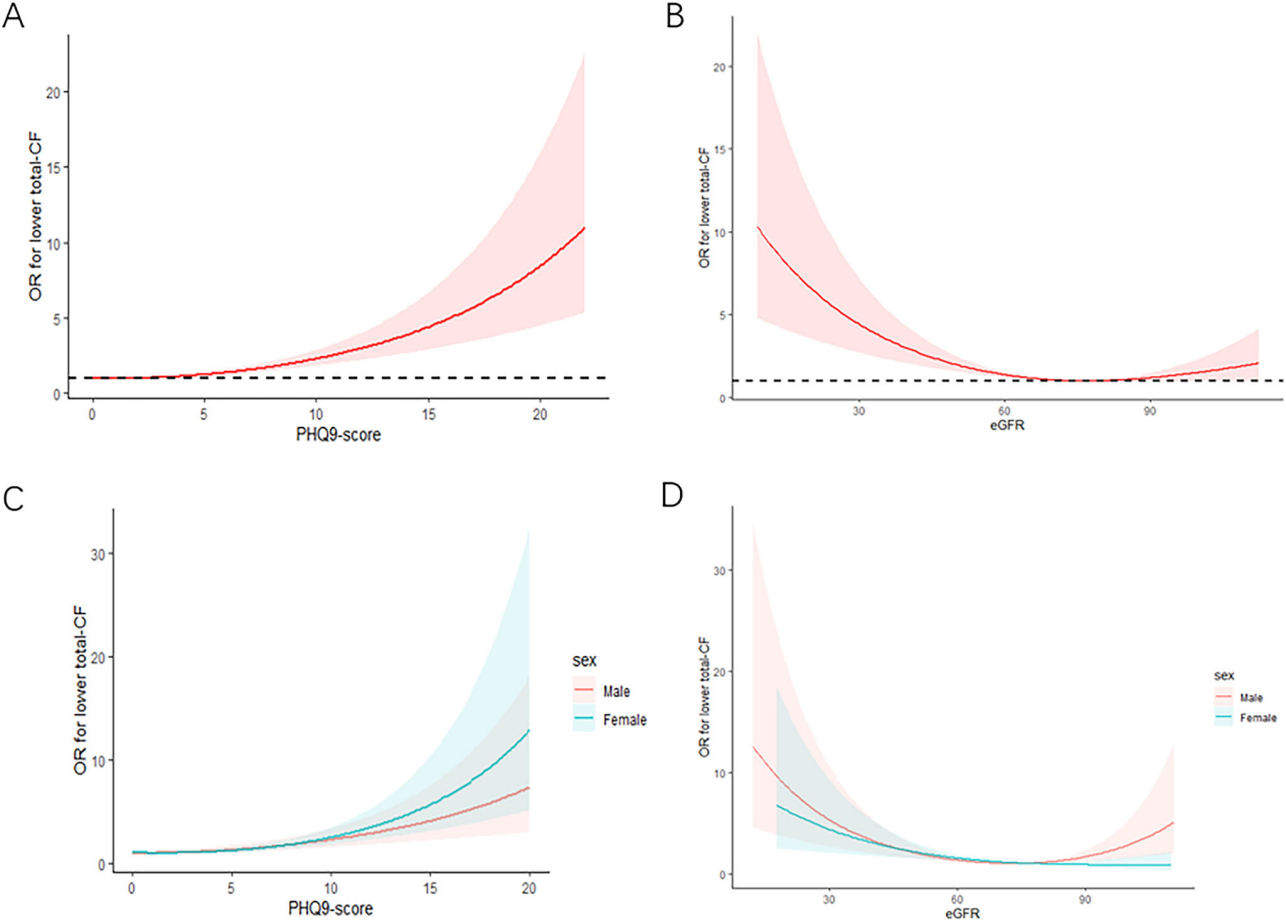
The restricted cubic spline (RCS) curves for the association between PHQ‐9 score, eGFR, and cognition decline, overall and stratified by sex. (A) Association between PHQ‐9 score and cognition decline risk. (B) Association between eGFR and cognition decline risk. (C) Sex‐stratified association between PHQ‐9 score and cognition decline risk. (D) Sex‐stratified association between eGFR and cognition decline risk.

Figure 2A depicts the RCS curve, highlighting a positive link between PHQ‐9 scores and cognition decline risk. The risk rises notably with increasing PHQ‐9 scores. Sex‐stratified analysis (Figure 2C) shows this association is more pronounced in females, while the correlation is weaker in males. As illustrated in Figure 2B, the relationship between eGFR and cognition decline risk follows an L‐shaped curve. For eGFR ≤ 77.04005 mL/min/1.73 m^2^, cognition decline risk declined significantly as eGFR increased (OR: 0.956, 95% CI: 0.939–0.974, *p* < 0.0001). Beyond this inflection point, the association was not statistically significant. Sex‐stratified analysis (Figure 2D) showed a stronger association in males, whereas the correlation was weaker in females.

### 3.3 Combined Association of Depressive Symptoms and eGFR With Cognition Decline Symptoms

The combined association of depressive symptoms and eGFR with cognition decline risk was evaluated. In the crude model, significant differences were observed for all groups relative to the reference group (without depressive symptoms and eGFR > 90). In Model 2, the adjusted ORs were: 2.60 (95% CI: 1.43–4.73, *p* = 0.01) for depressive symptoms and eGFR > 90; 2.62 (95% CI: 1.19–5.77, *p* = 0.02) for depressive symptoms and 60 < eGFR < 90; and 1.90 (95% CI: 1.06–3.39, *p* = 0.03) for without depressive symptoms and eGFR < 60. The highest cognition decline risk was found in the depressive symptoms and eGFR < 60 group, with an adjusted OR of 11.74 (95% CI: 4.02–34.28, *p* < 0.001) (Table 2C).

### 3.4 Subgroup Analysis

In individuals aged < 70 years, age‐stratified analysis showed that the combined impact of depressive symptoms and eGFR significantly heightened cognition decline risk. The high‐risk group (Q6) had an OR of 24.47 (95% CI: 5.05–118.72, *p* = 0.002), while groups Q2, Q4, and Q5 also showed significant increases. A trend test confirmed a significant rise in cognition decline risk across the groups (*p* = 0.004). In participants aged ≥ 70 years, the high‐risk group (Q6) exhibited a significantly increased cognition decline risk (OR = 13.35, 95% CI: 2.34–76.27, *p* = 0.009), with group Q5 showing a significant risk as well (*p* = 0.01). The trend test supported a gradual increase in cognition decline risk across the groups (*p* = 0.001).

The analysis indicated that females in the high‐risk group (Q6) had a significantly increased cognition decline risk (OR = 25.94, 95% CI: 6.22–108.21, *p* < 0.001), with groups Q2–Q5 also showing elevated risks and a significant upward trend (*p* < 0.001). Among males, cognition decline risk was significantly higher only in the high‐risk group (Q6) (OR = 6.77, 95% CI: 1.32–34.75, *p* = 0.027), and no significant overall trend was observed (*p* = 0.072). Interaction tests confirmed no significant interactions between sex or age groups (*p* for interaction > 0.05) (Table 3).

**TABLE 3:** Subgroup analyses of combined depressive symptoms and serum creatinine on cognition decline by age and sex.

Character	Q1	Q2	*p*/adjusted *p*	Q3	*p*/adjusted *p*	Q4	*p*/adjusted *p*	Q5	*p*/adjusted *p*	Q6	*p*/adjusted *p*	*p* for trend	*p* for interaction
Age													0.367
≥ 70	ref	0.89 (0.10, 8.28)	0.906/0.906	1.58 (0.92, 2.72)	0.089/0.116	2.92 (0.80, 10.70)	0.093/0.116	2.95 (1.41, 6.21)	0.01/0.025	13.35 (2.34, 76.27)	0.009/0.025	0.001	
< 70	ref	2.45 (1.25, 4.79)	0.015/0.037	0.92 (0.44, 1.93)	0.797/0.797	3.18 (1.10, 9.24)	0.037/0.051	3.51 (1.07, 12.10)	0.0410.051	24.47 (5.05, 118.72)	0.002/0.01	0.004	
Sex													0.098
Male	ref	1.48 (0.43, 5.06)	0.485/0.60	0.65 (0.37, 1.16)	0.127/0.31	1.55 (0.41, 5.94)	0.472/0.6	1.12 (0.50, 2.53)	0.751/0.75	6.77 (1.32, 34.75)	0.027/0.135	0.072	
Female	ref	5.33 (2.07, 13.77)	0.004/0.006	2.03 (1.17, 3.52)	0.018/0.018	5.14 (1.92, 3.75)	0.005/0.006	3.79 (1.71, 8.41)	0.005/0.06	25.94 (6.22, 108.21)	< 0.001/0.002	< 0.001	

*Note*: Q1: no depressive symptoms and eGFR > 90. Q2: depressive symptoms and eGFR > 90. Q3: no depressive symptoms and 60 < eGFR < 90. Q4: depressive symptoms and 60 < eGFR < 90. Q5: no depressive symptoms and eGFR < 60. Q6: depressive symptoms and eGFR < 60.

### 3.5 Mediation Analysis and Interaction

**FIGURE 3:**
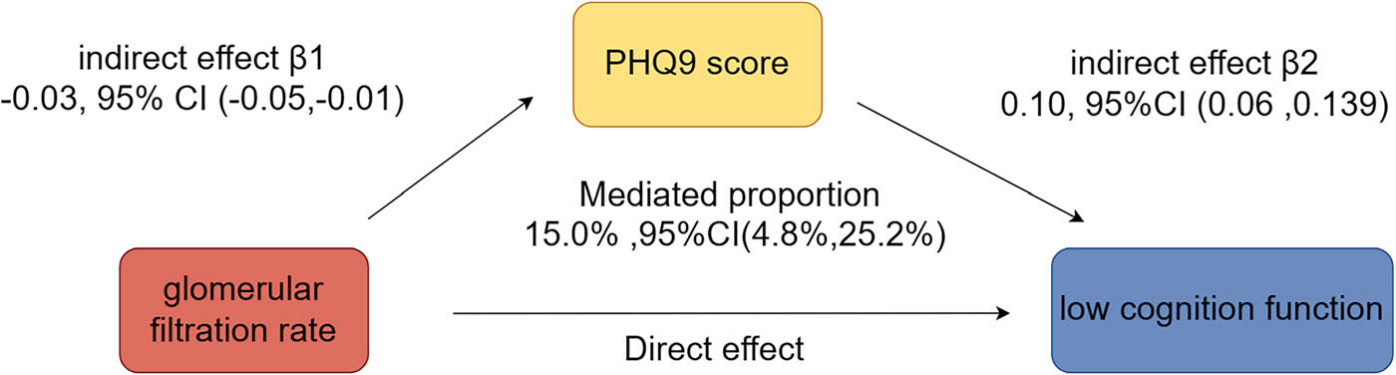
The mediating effect analysis of eGFR and cognition decline.

The mediation analysis assessed the role of eGFR in cognitive impairment and showed a significant mediating effect of PHQ‐9 scores on the eGFR‐cognitive impairment relationship, with a direct effect of 15% (95% CI: 4.8%–25.2%, *p* < 0.05) (Figure 3). No significant multiplicative or additive interaction was observed between PHQ‐9 scores and eGFR on cognitive decline. When testing higher‐order interactions, age × PHQ‐9 and age × eGFR were not significant, while sex × PHQ‐9 showed a modest but significant interaction (OR = 1.05, 95% CI: 1.00–1.10, *p* = 0.033), and sex × eGFR was borderline significant (OR = 0.99, 95% CI: 0.98–1.00, *p* = 0.082). These findings suggest that the impact of depressive symptoms on cognition may differ slightly by sex. (Table S1)

## 4 Discussion

This study, based on NHANES data, examined the individual and combined impacts of depressive symptoms and eGFR on the risk of developing cognition decline. The results demonstrated that both depressive symptoms and reduced eGFR significantly contribute to cognition decline risk, and their combined presence further amplifies this risk. These findings deepen our understanding of the interplay between depressive symptoms and renal dysfunction in the pathogenesis of cognition decline, while also highlighting the strengths of a large, population‐based design and multiple analytic approaches. Nonetheless, potential biases and methodological limitations—such as reliance on PHQ‐9 as a screening tool, limited cognitive test coverage, and residual confounding—should be considered when interpreting these results.

The relationship between depressive symptoms and cognition decline is well‐established, with depressive symptoms identified as both an early indicator and a contributing factor in accelerating the onset of cognitive decline. Depressive symptoms impair cognitive function through mechanisms including neuroinflammation, reduced cerebral blood flow, and neurotransmitter imbalances (Carrera‐González et al. 2022). The neuroinflammation hypothesis suggests that chronic depression elevates brain inflammatory markers, leading to neuronal damage and an increased risk of dementia (Ly et al. 2023), and this has been supported by experimental evidence showing that IL‐6 and TNF‐α elevations accelerate neuronal apoptosis and cognitive deficits in animal models (Diniz 2018; S. Liu, Fan, et al. 2022).

Furthermore, individuals with depression frequently adopt unhealthy lifestyles, such as physical inactivity, poor nutrition, and disrupted sleep—factors well‐recognized as increasing dementia risk (Dominguez et al. 2021). Compared with previous research, this study demonstrates that depressive symptoms significantly increase cognition decline risk when accompanied by low eGFR, highlighting a potential synergistic interaction between depression and renal dysfunction. eGFR, a critical indicator of kidney function, has been closely linked to cognitive decline (Chu et al. 2022). Renal dysfunction leads to toxin accumulation, vascular damage, and chronic inflammation, which collectively reduce cerebral blood flow and disrupt central nervous system function, ultimately accelerating cognitive decline (Pépin et al. 2023). Additionally, renal dysfunction induces metabolic disturbances and electrolyte imbalances, further compromising neural function (Dhondup and Qian 2017). Recent cohort studies have further shown that patients with coexisting CKD and depression have a substantially increased risk of developing dementia, providing clinical evidence consistent with our findings (T. Zhou et al. 2024). This study highlights that reduced eGFR significantly increases cognition decline risk, underscoring renal dysfunction as an independent and critical contributor to dementia pathogenesis.

Chronic depression often induces systemic inflammation, which is further aggravated by renal dysfunction, creating a heightened inflammatory state (Tinti et al. 2021). The intensified inflammation compromises the integrity of the blood–brain (BBB) barrier, allowing neurotoxic substances such as urea and creatinine to infiltrate the brain, damage neurons, and increase dementia risk (Xie et al. 2022). Experimental studies have confirmed that uremic toxins such as indoxyl sulfate aggravate hippocampal neuronal injury and cognitive impairment in animal models (Andrews et al. 2025). Oxidative stress plays a pivotal role in this interaction. Depression‐induced neurotransmitter disturbances and free radical activity, coupled with oxidative stress arising from renal dysfunction, exacerbate brain cell damage, accelerate neurodegeneration, and promote amyloid‐beta deposition, ultimately impairing cognitive function (Hussain et al. 2021). Vascular impairment is another crucial factor, as both depression and renal dysfunction are strongly linked to cardiovascular disease and vascular damage. These vascular abnormalities reduce cerebral blood flow and oxygen delivery, resulting in chronic ischemia and an increased risk of dementia (Elahi and Miller 2017). Declining endothelial function associated with these conditions further compromises BBB integrity, permitting the accumulation of neurotoxic substances in the brain and exacerbating cognitive decline (El Nekidy et al. 2021). Clinical neuroimaging studies also suggest that cerebral small vessel disease is more prevalent among patients with CKD and depression, which may represent an important vascular pathway linking the two conditions to cognitive decline (S. M. Liu, Zhang, et al. 2022; Krittanawong et al. 2023).

Research indicates that the combined effects of depression and renal dysfunction may differ among specific subgroups. Evidence suggests that female patients may exhibit greater vulnerability to depression's effects compared to males, potentially due to variations in hormone levels, inflammatory responses, and vascular health (Ivanets et al. 2021). As shown in our subgroup analyses (Table 3 and Figure 2D), the combined effect of depressive symptoms and reduced eGFR on cognitive decline was more pronounced in females than in males. Subgroup analysis also revealed that younger patients with concurrent depression and renal dysfunction are at a heightened risk of dementia, underscoring the critical need for early prevention and intervention in this population. Several biological and psychosocial mechanisms may underlie the stronger combined effect observed among females. Biologically, estrogen has neuroprotective and vasoprotective properties, and its decline after menopause is associated with heightened neuroinflammation and cerebrovascular vulnerability, potentially amplifying the cognitive impact of renal dysfunction and depressive symptoms (Ly et al. 2023; Bolego et al. 2010). Sex‐specific immune responses may further contribute; females often exhibit more robust inflammatory reactivity, and CKD is increasingly recognized as a systemic inflammatory state, which could magnify brain vulnerability when depressive symptoms co‐occur (Tinti et al. 2021; Voskuhl 2020). Psychosocial factors may also play a role, as women have a higher prevalence and reporting of depressive symptoms and may experience distinct stress exposures and role burdens, which can compound vascular–metabolic risks and cognitive trajectories (Arenaza‐Urquijo et al. 2024; Nebel et al. 2018). These interpretations remain hypothesis‐generating; prospective studies incorporating menopausal status, hormone therapy exposure, inflammatory biomarkers, and psychosocial measures are warranted to test these pathways. In addition, our formal interaction analyses indicated a modest but significant sex × PHQ‐9 interaction and a borderline sex × eGFR interaction, further supporting potential sex‐related differences in these associations.

Notably, some previous studies have reported significant combined effects of CKD and depression on cognitive impairment, such as the NHANES‐based analysis by T. Zhou et al. (2024). In contrast, our study did not detect a significant interaction between PHQ‐9 severity and eGFR. This discrepancy may be attributable to differences in population definitions (CKD vs. general population with varying eGFR levels), outcome classification, and analytic approaches, underscoring the need for further longitudinal studies.

Mediation analysis revealed that PHQ‐9 scores significantly mediated the association between reduced eGFR and cognitive impairment, indicating that depression may indirectly heighten dementia risk by exacerbating the impact of renal dysfunction on cognitive function. This finding aligns with the hypothesis that depression serves as a mediator linking renal dysfunction and cognitive decline, consistent with prior research (Zhou et al. 2024). However, interaction analysis revealed no significant interaction between PHQ‐9 scores and eGFR regarding their combined impact on cognition decline risk. This suggests that while both factors independently contribute to dementia risk, their combined effect appears additive rather than synergistic. These findings are consistent with previous research indicating that multiple health factors independently influence cognitive impairment (Ward et al. 2022). The gradual reduction of AIC values in both logistic and linear models indicates improved model fit after covariate adjustment, reinforcing the consistency of our findings across modeling approaches.

This study supports the hypothesis of a synergistic interaction between depression and reduced eGFR in increasing cognition decline risk, complementing prior research that independently associates these factors with cognition decline (Rubin 2018; Bugnicourt et al. 2013). Notably, this study is the first to identify the significant combined impact of depression and renal dysfunction on cognition decline risk. By employing a bivariate analysis approach, this study offers a novel perspective on the interplay of multiple health factors in cognition decline. Future research should further investigate the biological mechanisms linking depression, renal dysfunction, and cognition decline, which could inform the development of personalized prevention and management strategies. Additionally, exploring the role of other health factors, such as cardiovascular diseases and diabetes, may help build more comprehensive models for predicting cognition decline risk.

### 4.1 Limitation

This study has several limitations. First, depressive symptoms were assessed using the PHQ‐9, a screening rather than diagnostic instrument, which may cause some misclassification, and variations across cultural or ethnic groups could affect generalizability. Second, cognitive function was evaluated using a limited set of tests, which, although standardized in NHANES, may not fully capture all cognitive domains such as spatial or executive function. Third, residual confounding from unmeasured factors such as muscle mass and dietary protein intake cannot be excluded, which may influence eGFR estimates. Fourth, although multiple statistical analyses were conducted, we applied the Benjamini–Hochberg FDR correction across regression, subgroup, and interaction analyses to reduce the likelihood of Type I errors. However, given the relatively large number of comparisons, some findings should still be interpreted with caution and validated in independent cohorts. Finally, given the cross‐sectional design and baseline differences between groups, causal inference is precluded and selection bias may remain despite covariate adjustment. Future longitudinal and mechanistic studies are warranted to confirm these associations and to guide the development of targeted strategies for reducing the burden of cognitive decline.

## 5 Conclusion

The study underscores the important roles of depressive symptoms and renal dysfunction in relation to cognitive decline. Both depressive symptoms and reduced eGFR were independently associated with cognitive impairment, highlighting their relevance in population‐level research on dementia risk. Depressive symptoms may act as a mediator linking renal dysfunction to cognitive decline, suggesting the need for integrated approaches addressing both mental and renal health.

## Author Contributions

**Yuxin Yang**: data curation, investigation, writing–original draft, writing–review and editing, and validation. **Haoxiang Hu**: investigation, methodology, validation, writing–original draft and writing–review and editing. **Tian Lv**: data curation, formal analysis, methodology, writing–original draft, and software. **Jie Li**: investigation, formal analysis, writing–original draft, and visualization. **Yue Yang**: Methodology, Visualization, Validation, and Writing–review and editing. **Shiqin Chen**: Conceptualization, Methodology and Software, and Writing–review and editing. **Qinwen Fei**: Supervision, Conceptualization, Resources, and Writing–review and editing. **Yuping He**: Supervision, Project administration, Funding acquisition, and Writing–review and editing.

## Ethics Statement

The studies involving humans were approved by the NCHS Research Ethics Review Board at the National Center for Health Statistics. The studies were conducted in accordance with the local legislation and institutional requirements. Written informed consent for participation was not required from the participants or the participants’ legal guardians/next of kin in accordance with the national legislation and institutional requirements.

## Conflicts of Interest

The authors declare no conflicts of interest.

## Supporting information




**Table S1**: Interaction Analysis Results of PHQ‐9 Score and eGFR. (A) Interaction between PHQ‐9 and eGFR. (B) Additional interaction analyses with age and sex

**Table S2**: Sensitivity analysis using multivariable linear regression with composite cognitive score as a continuous outcome

**Table S3**: Model fit statistics (AIC) for logistic and linear regression analyses

## Data Availability

The original contributions presented in the study are included in the article/Supplementary material. The NHANES datasets analyzed in this study are publicly available from the Centers for Disease Control and Prevention (CDC) website: https://www.cdc.gov/nchs/nhanes/. Further inquiries can be directed to the corresponding authors.
