# Supplementary material for: Combined Influence of Depressive Symptoms and Estimated Glomerular Filtration Rate on Cognition Decline in US Adults
Source: Brain Behav. 2025 Nov 26;15(12):e70997. doi: 10.1002/brb3.70997 (PMC12657257; doi:10.1002/brb3.70997)
Supplement: Supplementary file 1 — Table S1: Interaction Analysis Results of PHQ‐9 Score and eGFR. (A) Interaction between PHQ‐9 and eGFR. (B) Additional interaction analyses with age and sex [file BRB3-15-e70997-s003.docx]

Table S1. Interaction analyses of depressive symptoms (PHQ-9) and eGFR, and additional interactions with age and sex, on cognitive decline

(A) Interaction between PHQ-9 and eGFR

| Category | Crude Model | Model 1 | Model 2 |
| --- | --- | --- | --- |
| PHQ-9 ＜10 and eGFR ＞60 | Ref | Ref | Ref |
| PHQ-9 ＜10 and eGFR ＜60 | 1.47 (1.28, 1.7) | 1.03 (0.86, 1.24) | 1.25 (1, 1.58) |
| PHQ-9 ≥10 and eGFR ＞60 | 3.14 (1.84, 5.38) | 3.65 (2.12, 6.3) | 3.01 (1.6, 5.66) |
| PHQ-9 ≥10 and eGFR ＜60 | 4.06 (2.71, 6.09) | 3.55 (2.35, 5.37) | 3.84 (2.34, 6.31) |
| Effect of eGFR (PHQ-9 ≥10) | 1.29 (0.92, 1.82) | 0.97 (0.68, 1.39) | 1.28 (0.83, 1.97) |
| Effect of PHQ-9 (eGFR ＜60) | 2.76 (1.91, 4) | 3.44 (2.34, 5.07) | 3.06 (1.95, 4.81) |
| Multiplicative Scale | 0.88 (0.61, 1.27) | 0.94 (0.65, 1.38) | 1.02 (0.66, 1.57) |
| RERI | 0.45 (-0.67, 1.56) | -0.13 (-1.44, 1.17) | 0.58 (-0.74, 1.9) |
| AP | 0.11 (-0.16, 0.38) | -0.04 (-0.4, 0.33) | 0.15 (-0.18, 0.48) |
| SI | 1.17(0.77, 1.77) | 0.95(0.59, 1.54) | 1.26(0.72, 2.19) |

Crude Model: adjusted for PHQ-9 and eGFR.

Model 1: adjusted for included crude model, age, sex, education and race.

Model 2: adjusted for all model 1 in addition to uric acid, NLR, protein, bilirubin, sleep hour, BMI, smoking status, alcohol status, hyperlipidemia and hypertension.

RERI: Relative Excess Risk due to Interaction; AP: Attributable Proportion due to Interaction; SI: Synergy Index

(B) Additional interaction analyses with age and sex

| Interaction | OR (95%CI) | P value |
| --- | --- | --- |
| Age × PHQ-9 | 1.001 (0.998, 1.004) | 0.56 |
| Sex × PHQ-9 | 0.9997 (0.999, 1.000) | 0.47 |
| Age × eGFR | 1.049 (1.004, 1.097) | 0.033 |
| Sex × eGFR | 0.991 (0.981, 1.001) | 0.082 |

Multiplicative interaction terms were tested in logistic regression models adjusted for covariates in Model 2.

Interaction effects: Multiplicative interaction tests whether the joint effect differs from the product of individual effects, while additive interaction tests whether the joint effect differs from the sum of individual effects. Significant interaction therefore indicates departure from simple additivity or multiplicativity.
