# Supplementary material for: Combined Influence of Depressive Symptoms and Estimated Glomerular Filtration Rate on Cognition Decline in US Adults
Source: Brain Behav. 2025 Nov 26;15(12):e70997. doi: 10.1002/brb3.70997 (PMC12657257; doi:10.1002/brb3.70997)
Supplement: Supplementary file 2 — Table S2: Sensitivity analysis using multivariable linear regression with composite cognitive score as a continuous outcome [file BRB3-15-e70997-s001.docx]

Table S2. Sensitivity analysis using multivariable linear regression with composite cognitive score as a continuous outcome

| Exposure | Crude Model | | Model 1 | | Model 2 | |
| --- | --- | --- | --- | --- | --- | --- |
|  | 95%CI | P | 95%CI | P | 95%CI | P |
| PHQ-9 score | -0.043(-0.058, -0.027) | <0.0001 | -0.038(-0.050, -0.025) | <0.0001 | -0.035 (-0.047, -0.022) | <0.0001 |
| eGFR | 0.012(0.009,0.015) | <0.0001 | 0.003(0.001, 0.005) | 0.016 | 0.004 (0.001, 0.006) | 0.010 |

Crude model: included only the main predictor (either PHQ-9 score or eGFR).

Model 1: adjusted for the main predictor, age, sex, and education.

Model 2: adjusted for the main predictor, age, sex, education, smoking status, alcohol use, neutrophil-to-lymphocyte ratio (NLR), hypertension, hyperlipidemia, BMI, uric acid, protein, bilirubin, and sleep duration.
