# Supplementary material for: Combined Influence of Depressive Symptoms and Estimated Glomerular Filtration Rate on Cognition Decline in US Adults
Source: Brain Behav. 2025 Nov 26;15(12):e70997. doi: 10.1002/brb3.70997 (PMC12657257; doi:10.1002/brb3.70997)
Supplement: Supplementary file 3 — Table S3: Model fit statistics (AIC) for logistic and linear regression analyses [file BRB3-15-e70997-s002.docx]

Table S3. Model fit statistics (AIC) for logistic and linear regression analyses

| Exposure–Outcome | Model | Logistic regression (binary outcome: cognitive decline) AIC | Linear regression (continuous outcome: cognitive score) AIC |
| --- | --- | --- | --- |
| eGFR → cognition | Crude model | 1695 | 8351 |
|  | Model 1 | 1426 | 7252 |
|  | Model 2 | 1393 | 7059 |
| PHQ-9 → cognition | Crude model | 1745 | 8400 |
|  | Model 1 | 1391 | 7163 |
|  | Model 2 | 1368 | 6989 |

Crude model: unadjusted.

Model 1: adjusted for the main predictor, age, sex, and education.

Model 2: adjusted for the main predictor, age, sex, education, smoking status, alcohol use, neutrophil-to-lymphocyte ratio (NLR), hypertension, hyperlipidemia, BMI, uric acid, protein, bilirubin, and sleep duration.
